# Moxibustion for diarrhea in children: A protocol for systematic review and meta-analysis

**DOI:** 10.1097/MD.0000000000025712

**Published:** 2021-04-30

**Authors:** Peiling Li, Zhenhai Chi, Jianyu You, Gen Deng, Xingchen Zhou, Qiangjian Mao, Zefeng Pan

**Affiliations:** a Jiangxi University of Traditional Chinese Medicine; b Affiliated Hospital of Jiangxi University of Traditional Chinese Medicine, China.

**Keywords:** children, diarrhea, moxibustion, random, systematic review and meta-analysis

## Abstract

**Background:**

Infantile Diarrhea is a common and frequent digestive tract disease in children. The causes of this disease are relatively complex and the onset time is relatively long. At present, there is no specific treatment method in Western medicine. Moxibustion is a simple and painless external treatment. However, due to the lack of high-quality evidence to support the effectiveness and safety of moxibustion therapy for pediatric diarrhea. Therefore, the purpose of this study is to verify the effectiveness and safety of moxibustion in the treatment of pediatric diarrhea.

**Methods:**

We will use PubMed, Cochrane Library, Wan Fang Database, Web of Science, China National Knowledge Infrastructure Database, Chinese Science Journal Database, China Biomedical Literature Database to carry out a progressive search of diseases. The study will be screened according to eligibility criteria, and quality of the study will be assessed by using the Cochrane Risk of Bias Tool.

**Results:**

Through this study, we will systematically evaluate the effectiveness and safety of moxibustion in the treatment of pediatric diarrhea.

**Conclusion:**

The results of this study will provide reliable evidence of the safety and effectiveness of moxibustion in the treatment of infantile diarrhea, and provide a therapeutic basis for the future clinical application.

**Ethics and dissemination:**

Since this paper does not involve ethical issues, it does not need to pass the review of the ethics committee. It can only collect relevant literature and study.

**INPLASY Registration number:**

INPLASY202130091

## 1 Introduction

Infantile diarrhea is a digestive tract syndrome caused by multiple pathogens and factors. Its clinical manifestations are increased stool frequency, or changes in the nature and shape of stool, as well as feces containing substances such as mucus, pus, and blood.^[1,2]^ Long-term diarrhea is prone to dehydration, electrolyte disturbance and other symptoms, and too long duration can cause growth retardation, and even death in children.^[3,4]^ According to records, children who die of diarrhea every year in the world are mostly due to dehydration and electrolyte disturbance caused by diarrhea.^[5,6]^ At present, most clinical drugs are mainly used to protect intestinal mucosa, intestinal microecological agents and prevent dehydration, and side effects will inevitably occur in the process of drug use.^[7]^ Therefore, it is necessary to seek a safer and more effective alternative therapy.

Traditional Chinese medicine deem that the disease site of diarrhea in children is in the spleen, stomach, and large intestine, dysfunction of the spleen in transportation is the key, and invigorating spleen for eliminating dampness is the basic principle of the treatment of diarrhea.^[8]^ According to modern medical research, moxibustion is an external treatment that stimulates the regulation system in the body by burning moxa, makes the maladjusted function operate normally, and has the effects of warming the meridian and dissipating cold, promoting blood circulation and dredging collateral, preventing disease and health care, etc.^[9]^ In addition, moxibustion therapy can also promote gastrointestinal peristalsis and repair of gastrointestinal mucosa while inhibiting intestinal hypermotility through bidirectional regulation.^[9,10]^ A large number of studies have proved that moxibustion, as an external treatment of traditional Chinese medicine, can not only make the children do not resist at the same time, but also make the effect of the exact.

## 2 Methods

### 2.1 Study registration

This Agreement was registered on the International Platform for Registrar System Review and Meta-Analysis Protocols (INPLASY) on March 24, 2021 and last updated on March 24, 2021 (Registration No. INPLASY202130091).

### 2.2 Database

We will search PubMed, Web of Science, Cochrane Library, China National Knowledge Infrastructure (CNKI), Wanfang Database, China Scientific Journals Database (VIP), and China Biomedical Literature Database (CBM) databases. The included literatures are limited to Chinese and English publications, and the time limit will be January 31, 2021.

### 2.3 Search strategy

Moxibustion, Infantile, diarrhea, and random were used as the key words, and the method of “subject word + free word” was adopted to conduct the retrieval on PubMed. Table 1 shows a draft search strategy using PubMed.

**Table 1:** PubMed database search policies.

ID	Search
#1	“Diarrhea”[Mesh]
#2	(((Diarrheas[Ti/Ab]) OR (loose bowels[Ti/Ab])) OR (Explosive diarrhea[Ti/Ab])) OR (Chronic diarrhea[Ti/Ab])
#3	#1 OR #2
#4	“Infantile”[Mesh]
#5	((Pediatric[Ti/Ab]) OR (Childeren [Ti/Ab])) OR (Child[Ti/Ab])
#6	#4 OR #5
#7	“Moxibustion”[Mesh]
#8	((Needle warming moxibustion[Ti/Ab]) OR (Heat-sensitive moxibustion[Ti/Ab])) OR (Traditional moxibustion[Ti/Ab])
#9	#7 OR #8
#10	((Random[Ti/Ab]) OR (Controlled trial[Ti/Ab])) OR (RCT[Ti/Ab])
#11	#3 AND #6 AND #9 AND #10

### 2.4 Inclusion and exclusion criteria

#### 2.4.1 Types of studies

Only publications in Chinese and English as the first language were included in this study, and only randomized controlled trials were included. The experimental group was moxibustion therapy or moxibustion combined with other therapies, and the control group was other therapies except moxibustion therapy. Other designs such as non-randomized controlled trials, animal studies, individual cases, and reviews will be excluded.

#### 2.4.2 Types of participants

All the children included were diagnosed with diarrhea, regardless of gender, age, race, nationality, or severity. The inclusion criteria in China were based on the “Chinese Diarrhoeal Disease Diagnosis and Treatment Program” revised by the Ministry of Health of the People's Republic of China in 1998.^[11]^

#### 2.4.3 Types of intervention

The moxibustion methods in the experimental group could be traditional moxibustion, heat-sensitive moxibustion, needle warming moxibustion and other types of moxibustion therapy. The control group received one of the following treatments: conventional drug treatment, no treatment, or placebo.

#### 2.4.4 Types of outcomes

The curative effect such as cure rate, apparent efficiency, effective rate, and inefficiency will be the main evaluation indexes.

### 2.5 Data extraction and analysis

#### 2.5.1 Data extraction

**Figure 1:**
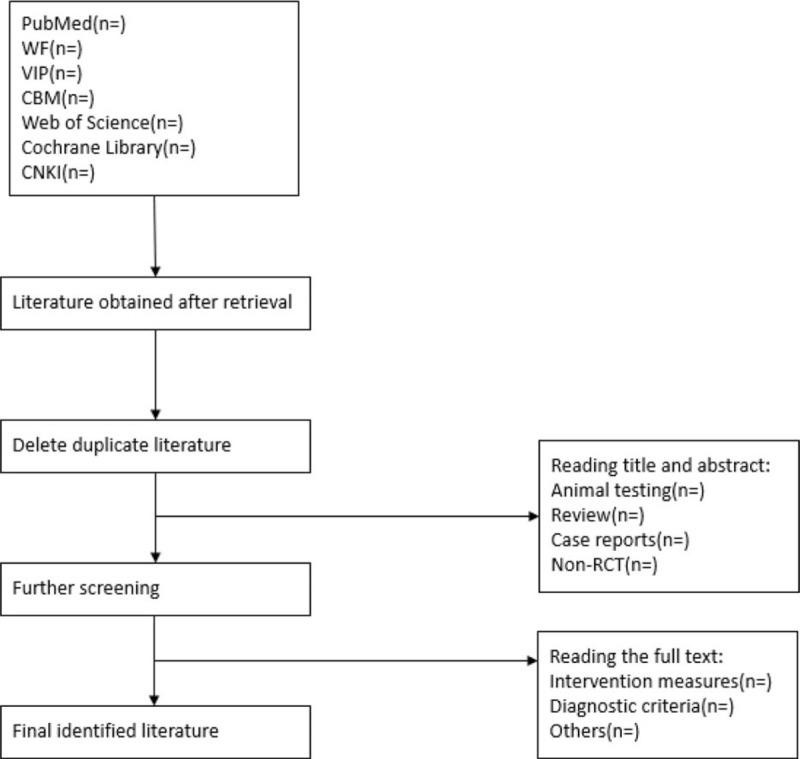
Flowchart of literature selection.

The titles and abstracts of the included studies were reviewed independently by 2 investigators, and the full texts were read if necessary. The studies were screened for study design, interventions, methods, the index of measures, results, and methodological content such as hidden grouping and blind method. If there is any disagreement, the data will be verified by a third investigator. If the literature information is incomplete, the original author will be contacted to obtain the data. Figure 1 is the flow chart of preliminary screening.

#### 2.5.2 Risk assessment of the included literature

The authors use the Cochrane collaboration of bias risk assessment tools to evaluate each other law quality, and the bias risk assessment included in the study are available from the following aspects: the formation of sequence, allocation concealment, the result data is imperfect, the interpretation of the results, the wrong data coding, and other bias. The bias can be divided into 3 risks: high risk, low risk, and uncertain risk.

### 2.6 Summary statistics

#### 2.6.1 Heterogeneity analysis

*I*^*2*^ test was used to test the heterogeneity of the study. If *P* > .10 and *I*^*2*^ ≤ 50%, no statistical difference is considered, fixed effect model will be used. If *P* ≤ .10 and *I*^*2*^ > 50%, the heterogeneity is considered to be high, and the random effect model will be adopted.

#### 2.6.2 Publication bias

RevMan 5.4 was used to draw funnel plots to detect publication bias. When the funnel plots were symmetrical, it was considered that there was no publication bias; when the scatter points on the funnel plots were asymmetrical, it was the other way around.

#### 2.6.3 Subgroup analysis

In the case of sufficient literature data, subgroup analysis can be conducted according to the severity of diarrhea or the intervention mode of moxibustion therapy.

1. The severity of diarrhea (such as duration, frequency of stools, associated symptoms, etc.)
2. Types of moxibustion (such as Heat-sensitive moxibustion, needle warming moxibustion, traditional moxibustion, etc.).

#### 2.6.4 Sensitivity analysis

According to the outcome indicators of the data, literatures were excluded one by one in terms of method quality, sample size, data missing, measurement, etc to see whether the heterogeneity was changed. If the results of the sensitivity analysis are consistent, the results are relatively robust. On the contrary, it is not robust and should be treated with caution.

## 3 Discussion

Diarrhea in children is a common clinical digestive tract disease, which can occur throughout the year, especially in summer and autumn. Its clinical manifestations are mainly characterized by increased stool frequency, thin stools, failure to dissipate grains, or even watery symptoms.^[11,12]^ The causes of its occurrence are mainly related to factors such as irregular diet, perception of exogenous pathogenic factors, spleen, and stomach weakness .Children's constitution is delicate, vulnerable to the invasion of disease evil, injury to the diet, spleen and stomach weakness, and the spleen and stomach transport dysfunction.^[13]^ As a category of external treatment, moxibustion therapy of traditional Chinese medicine has the characteristics of simple operation, convenient use, significant curative effect and less toxic and side effects, and plays the role of treating diseases, enhancing physical fitness, and preventing diseases before diseases.^[14,15]^ Western medicine in the treatment of pediatric diarrhea in the clinical use of gastrointestinal mucosal protectant, antibiotics and other drugs, the effect is more limited but also with certain side effects.^[16,17]^ The process of medication is also facilitated by making children resist. Therefore, it is of great significance to explore moxibustion as a safe and effective external therapy that is easy to be accepted by children and their parents.

## Author contributions

**Conceptualization:** Zhenhai Chi.

**Data curation:** Peiling Li, Gen Deng.

**Formal analysis:** Peiling Li, Jianyu You.

**Investigation:** Peiling Li, Gen Deng.

**Methodology:** Gen Deng, Qiangjian Mao.

**Project administration:** Zhenhai Chi, Jianyu You.

**Resources:** Jianyu You.

**Software:** Jianyu You, Gen Deng.

**Supervision:** Zhenhai Chi, Jianyu You.

**Validation:** Xingchen Zhou.

**Visualization:** Qiangjian Mao, Zefeng Pan.

**Writing – original draft:** Peiling Li, Zhenhai Chi.

**Writing – review & editing:** Peiling Li, Zhenhai Chi.
